# Extracellular Sphingomyelinase Rv0888 of *Mycobacterium tuberculosis* Contributes to Pathological Lung Injury of *Mycobacterium smegmatis* in Mice *via* Inducing Formation of Neutrophil Extracellular Traps

**DOI:** 10.3389/fimmu.2018.00677

**Published:** 2018-04-04

**Authors:** Guanghui Dang, Yingying Cui, Lei Wang, Tiantian Li, Ziyin Cui, Ningning Song, Liping Chen, Hai Pang, Siguo Liu

**Affiliations:** ^1^State Key Laboratory of Veterinary Biotechnology, Division of Bacterial Diseases, Harbin Veterinary Research Institute, Chinese Academy of Agricultural Sciences, Harbin, China; ^2^School of Medicine, Tsinghua University, Beijing, China

**Keywords:** *Mycobacterium tuberculosis*, sphingomyelinase, Rv0888, neutrophil extracellular traps, lung injury

## Abstract

*Mycobacterium tuberculosis* is the causative agent of tuberculosis (TB), which mainly causes pulmonary injury and tubercles. Although macrophages are generally considered to harbor the main cells of *M. tuberculosis*, new evidence suggests that neutrophils are rapidly recruited to the infected lung. *M. tuberculosis* itself, or its early secreted antigenic target protein 6 (ESAT-6), can induce formation of neutrophil extracellular traps (NETs). However, NETs trap mycobacteria but are unable to kill them. The role of NETs’ formation in the pathogenesis of mycobacteria remains unclear. Here, we report a new *M. tuberculosis* extracellular factor, bifunctional enzyme Rv0888, with both nuclease and sphingomyelinase activities. Rv0888 sphingomyelinase activity can induce NETs’ formation *in vitro* and in the lung of the mice and enhance the colonization ability of *Mycobacterium smegmatis* in the lungs of mice. Mice infected by *M. smegmatis* harboring Rv0888 sphingomyelinase induced pathological injury and inflammation of the lung, which was mainly mediated by NETs, induced by Rv0888 sphingomyelinase, associated protein (myeloperoxidase) triggered caspase-3. In summary, the study sheds new light on the pathogenesis of mycobacteria and reveals a novel target for TB treatment.

## Introduction

*Mycobacterium tuberculosis* is the pathogenic agent of tuberculosis (TB) (1), which is transmitted from person to person *via* tiny droplets from coughs or sneezes. In 2016, it is reported that there were about 10.4 million new TB instances and 1.7 million TB deaths world-wide (2). Although the number of TB deaths fell by 37% between 2000 and 2016, it continued to be ninth leading cause of decease world-wide in 2016 (2). The fundamental features of *M. tuberculosis* infection are the generation of pulmonary injury and tubercles (3). Previous researches have demonstrated that the first immune reaction is the movement of neutrophils to the location of infection, and the center of the lung granulomas is filled by neutrophils during mycobacterial infection, which appears to be the most important step during the TB (4–6). Furthermore, human neutrophils were capable to phagocytose *M. tuberculosis in vitro*, although they failed to kill the bacilli (7).

Neutrophils are the most plentiful leukocytes in human blood and respond rapidly to infection and acute inflammation (8). While tissues are invaded *via* bacterial or fungal pathogens, neutrophils are recruited to the infection site swiftly (9). After recruitment to the inflammatory site, entering pathogens are attacked by neutrophils through release of lytic enzymes and antimicrobial peptides as well as by production of reactive oxygen species (ROS); this is followed through phagocytosis, which promotes clearance of the invading microorganisms (10–12). Neutrophils’ another reported antimicrobial mechanism is the formation of neutrophil extracellular traps (NETs) (13), which are consisted of DNA/histones, granular proteins such as neutrophil elastase and myeloperoxidase (MPO), and cytoplasmic proteins (14). Various stimuli such as interleukin-8, lipopolysaccharide or phorbol myristate acetate (PMA) (13), bacteria (15, 16), fungi (17), viruses (18), parasites (19), α-enolase of *Streptococcus pneumoniae* (20), and mycoplasma lipoproteins (21) induce formation of NETs. These structures reportedly play a beneficial role in host defense mechanisms against pathogens, contributing to trap and destroy bacteria, fungi, and protozoa both *in vitro* and *in vivo* (22). However, excessive NETs can lead to inflammatory conditions, including atherosclerosis, vascular disorders, arthritis, sepsis, venous thrombosis, tumor metastasis, lung inflammation, and pneumonia (14, 23, 24). In addition, histones and MPO associated with NETs can cause epithelial and endothelial cell damage (25). Specifically, MPO activity can induce damage to adjacent tissue and cause various inflammatory diseases, including pulmonary injury (26, 27). Extracellular histones have also shown *in vitro* cytotoxicity toward the endothelium (28).

Two types of *M. tuberculosis* (H37Rv and *Mycobacterium canetti*) stimulate neutrophils to release NETs coated with neutrophil elastase and histones (29). In addition, NETs have the ability to capture mycobacteria but not kill them *in vitro* (29), which may favor lung destruction in active TB. Early secreted antigenic target protein-6 (ESAT-6), a protein secreted by *M. tuberculosis*, induces the production of NETs colocalized with MPO (3). Most of the studies on NETs induced by *M. tuberculosis* (29, 30) have been performed *in vitro* using primary peripheral neutrophils separated from blood. However, the formation of NETs has not been reported *in vivo*, and the functions of NETs in lung injury have not been clearly established.

Previous studies have demonstrated that extracellular Rv0888 of *M. tuberculosis* is a bifunctional enzyme with both nuclease (31) and sphingomyelinase (32) activities. In this study, we found that Rv0888 sphingomyelinase activity induced the formation of NETs *in vitro* and in the lungs of mice infected by recombinant *Mycobacterium smegmatis*. The protein components of the formed NETs, especially MPO, triggered pathological lung injury through the caspase-3 pathway.

## Materials and Methods

### Animals, Bacterial Strains, and Growth Conditions

The C57BL/6 mice (6–8 weeks old) used in this study were purchased from Chang Sheng Biotechnology (Liaoning, China). All of the experimental work that involved animals was carried out following the instructions that were suggested by the Heilongjiang Animal Ethics Committee at the Heilongjiang science and technology government agency (Harbin, China) and was approved and supervised by the Commissioner for Animal Welfare at the Harbin Veterinary Research Institute, representing the Institutional Animal Care and Use Committee. All of the experimentations were conceived to minimize the numbers of animals that were used, and effort was made to minimize both distress and their pain. *M. smegmatis* MC2 155 cultures were grown in Middlebrook 7H9 medium (BD Biosciences, San Jose, CA, USA) and supplemented with 0.05% Tween 80 (Amresco, Solon, OH, USA) and 0.2% glycerol (Sigma-Aldrich, Shanghai, China).

### Preparation of Recombinant *M. smegmatis*

Recombinant *M. smegmatis* (pMV262/MS and Rv0888NS/MS) was prepared as described in a previous study (31). Briefly, the pMV262-Rv0888NS template plasmid was amplified using complementary mutagenic oligonucleotide pairs (Table S1 in Supplementary Material) to introduce nuclease mutant D438A (31) and sphingomyelinase mutant H481N (32) substitutions into the nucleotide sequence. PCR amplification was accomplished with PrimeSTAR Max DNA Polymerase (TaKaRa Bio, Beijing, China). The PCR products were digested using *Dpn*I to make the methylated parental template DNA damaged and DH5α *E. coli* were transformed with the mutated plasmids. All of the substitutions were identified by DNA sequencing. The recombinant plasmids (pMV262-D438A and pMV262-H481N) were transformed into *M. smegmatis* MC2 155 cells, and the recombinant *M. smegmatis* were named D438A/MS and H481N/MS, respectively.

### Expression and Purification of Rv0888NS, D438A, and H481N in *M. smegmatis*

The recombinant pMV262/MS, Rv0888NS/MS, D438A/MS, and H481N/MS were cultured in 7H9 broth medium (containing 0.05% Tween 80, 0.2% glycerol, and 50 µg/ml kanamycin) at 37°C until the OD600 reached 0.8–1.0. Protein expression was induced for 24 h at 42°C and was analyzed by Western blotting with monoclonal anti-Rv0888 antibody (4E6). The expressed Rv0888NS, D438A, and H481N were purified using previously described methods (31). The collected proteins were analyzed by SDS-PAGE.

### Localization of the Rv0888NS, D438A, and H481N Proteins

Preparation of subcellular fractions from the recombinant Rv0888NS/MS, D438A/MS, and H481N/MS was performed as described previously (33). Briefly, a 50 ml culture of recombinant *M. smegmatis* was centrifuged at 12,000 × *g* for 20 min at 4°C, and the supernatant was filtered using a 0.22-µm pore membrane to generate the culture filtrate fraction. Then, the pellet was resuspended in PBS containing a DNase, RNase, and proteinase inhibitor cocktail (Roche, Shanghai, China). Cells were sonicated for 30 min in an ice bath. The sonicate was centrifuged at 3,000 × *g* for 10 min at 4°C, and the supernatant was then centrifuged at 27,000 × *g* for 1 h at 4°C. The supernatant was collected for further processing, and the pellet was resuspended in PBS containing lysozyme, incubated at 37°C for 50 min at 100 rpm, and centrifuged at 27,000 × *g* for 1 h at 4°C. The pellet was resuspended in 10 mM ammonium bicarbonate and was labeled as the cell wall. The supernatant was blended with the fraction gathered from the previous centrifugation and was centrifuged at 100,000 × *g* for 4 h at 4°C. The final supernatant was marked as the cytosolic fraction, and the pellet was marked as the membrane protein fraction. The fractions were used in all of the immunodetection assays.

### Determining the Activity of the Rv0888NS, D438A, and H481N Proteins

To measure the DNase activity of the recombinant proteins Rv0888NS, D438A, and H481N, 0.2 µg of circular plasmid DNA (pGEX-6p-1 vector) was incubated with 3 µg of the purified Rv0888NS, D438A, or H481N protein in 10 µl of reaction buffer (20 mM Tris–HCl, 5 mM CaCl_2_, 5 mM MnCl_2_, pH 6.5) at 41°C. Buffer (20 mM Tris–HCl, pH 6.5) served as a negative control. After 60 min, 10 µl of the reaction solution combining with 1 µl of 10× loading buffer were loaded and analyzed by electrophoresis on a 1.0% agarose gel. To determine the sphingomyelinase activity of the recombinant proteins Rv0888NS, D438A, and H481N, the Amplex Red Sphingomyelinase Assay Kit (Molecular Probes, USA) was used according to the manufacturer recommendations. Sphingomyelinase of *Bacillus cereus* with known activity was used as a positive control.

### Detection of NETs *In Vitro*

Human neutrophils were separated from heparinized blood, obtained from healthy donors, with a Human Neutrophils Separation Medium Kit (TBD Science, Tianjin, China) according to the manufacturer’s instructions. The neutrophils were washed twice in RPMI 1640 medium (Thermo Scientific, Waltham, MA, USA) and resuspended in RPMI 1640 supplemented with 10% FBS (Thermo Scientific) for NETs assays.

Neutrophils were seeded at 2 × 10^5^ per Class bottom cell culture dishes (15 mm NEST, USA) pre-treated with 25 µg/ml of poly-l-lysine (Sigma-Aldrich) in 1 ml and allowed to settle for 30 min at 37°C in a 5% CO_2_ incubator. To observe the formation of NETs, neutrophils were stimulated with 0.4 or 0.8 mM recombinant proteins (Rv0888NS, D438A, and H481N in 50 mM Tris–HCl, pH 7.2, 10 mM MgCl_2_, 0.66 mM CaCl_2_, and 100 mM NaCl) for 4 h. Neutrophils were treated with 100 nM PMA (Sigma-Aldrich) to induce the formation of NETs as a positive control. The cells were fixed with 4% paraformaldehyde for 30 min and blocked for 2 h with 10% goat serum (Boster, China), 5% fish gelatin (Sigma-Aldrich), and 1% bovine serum albumin (Amresco, USA) at room temperature (RT). Anti-DNA/histone H1 antibody (mouse, 1:100, Merck Millipore, USA) and anti-MPO antibody (rabbit, 1:800, Abcam, ab139748) were added, and the cells were incubated overnight at 4°C followed by incubation with donkey anti-mouse IgG (Alexa Fluor 488) (1:500, Abcam, ab150105) and goat anti-rabbit IgG (Alexa Fluor 633) (1:200 ThermoFisher Scientific) for 1 h at RT. DNA was detected by the addition of DAPI (5 µg/ml, Sigma-Aldrich). Specimens were mounted in 50% glycerol and analyzed with a Leica TCS SP5 confocal laser-scanning microscope.

For quantification of DNA release, 200 µl of human neutrophils (2 × 10^5^/ml) was seeded in 96-well black plates and allowed to settle for 30 min at 37°C in a 5% CO_2_ incubator. The neutrophils were activated with 0.4 or 0.8 mM recombinant proteins (Rv0888NS, D438A, and H481N) and incubated for 4 h at 37°C in 5% CO_2_; 100 nM PMA was used as a positive control. A 0.5 µM quantity of Sytox Green, a non-cell-permeable DNA-binding dye, was added, and the cells were incubated for 10 min. Fluorescence was measured in a multimode microplate reader (EnSpire, PerkinElmer, USA) at an excitation wavelength of 485 nm and an emission wavelength of 527 nm. Three replicates per treatment were performed.

### Infection and Inhibitor Assays *In Vivo*

The C57BL/6 mice (*n* = 3 per group) were treated intraperitoneally with 2 × 10^6^ recombinant Rv0888NS/MS, D438A/MS, and H481N/MS in 100 µl of PBS with 0.05% Tween 80 (PBST). Control mice were treated with PBS and recombinant pMV262/MS. Co-injection of the histone inhibitor polysialic acid (PLA, 125 mg/kg body weight; Sigma-Aldrich), caspase-3 inhibitor (Z-DEVD-FMK, 3.4 mg/kg body weight; Abcam), and the MPO inhibitor (90 mg/kg body weight, Abcam) was performed in mice (*n* = 3) challenged with recombinant Rv0888NS/MS. These inhibitors follow the standard biosecurity of the Safety Data Sheet of the Abcam or Sigma-Aldrich.

### Detection of the Colonization of Recombinant *M. smegmatis* in Mice Lung and Analysis of the Level of the ROS and Ceramide in Infected Mice

At 1, 4, 7, 14, and 21 days after infection, three mice from each group were killed. The whole left lobe of the lung was homogenized with aseptic LB and then spread out onto LB agar plates that contained 50 µg/ml kanamycin. The plates were incubated at 37°C for 3 days for bacterial counting. After 2 days following administration of recombinant pMV/262/MS, Rv0888NS/MS, D438A/MS, and H481N, the mice were anesthetized with diethyl ether, and the eyeballs of the mice were removed to obtain the blood followed by serum separation. Then, the mice were killed, the left lungs were removed, and the lung homogenates were obtained with LB. The Mouse ROS enzyme-linked immunosorbent assay (ELISA) Kit and Mouse Ceramide ELISA Kit (Y-Y Chemical Reagents, Shanghai, China) were used to determine the levels of ROS and ceramide in the serum and pulmonary homogenates among the treatments.

### Detection of NETs *In Vivo*

The mice were injected intraperitoneally with recombinant Rv0888NS/MS, D438A/MS, or H481N/MS. Control mice were treated with PBS and recombinant pMV262/MS. After 48 h, the mice were killed, and the right lungs were removed. Frozen 6-µm lung tissue sections were fixed in ice-cold acetone for 15 min. The Vector M.O.M. Immunodetection Kit (Vector Laboratories, Burlingame, CA, USA) was used with some modifications. Briefly, after blocking the sections in M.O.M. Mouse Ig Blocking Reagent at RT for 1 h, samples were incubated with primary anti-MPO antibody (mouse, 1:50, Abcam, ab90810) or anti-histone H3 (citrulline R2 + R8 + R17) antibody (rabbit, 1:400, Abcam, ab5103), followed by incubation with a secondary goat anti-rabbit IgG antibody (Alexa Fluor 488) (1:500, Abcam) and a goat anti-mouse IgG antibody (Alexa Fluor 633) (1:200 ThermoFisher Scientific). Slices were mounted in Fluoroshield with DAPI (Sigma-Aldrich) and analyzed with a Leica TCS SP5 confocal laser-scanning microscope.

### Bronchoalveolar Lavage Fluid (BALF) Collection and Western Blot Analysis

Mice infected by recombinant pMV262/MS, Rv0888NS/MS, D438A/MS, and H481N/MS were anesthetized with diethyl ether, and the trachea was exposed. Subsequently, a closed IV catheter system (BD Intima, Franklin Lakes, NJ, USA) was inserted into the trachea and washed with 500 µl PBS three times. The collected BALF was incubated with 100 U DNase I (Amresco, Solon, OH, USA) at 37°C for 30 min, and the supernatant was analyzed by Western blotting with the following primary antibodies: anti-histone H4 (Abcam, ab17036), anti-histone H3 (citrulline R2 + R8 + R17) (Abcam), and anti-MPO (Abcam, ab139748). Then, incubation with secondary anti-mouse IgG Dylight 680-labeled antibody or anti-rabbit IgG Dylight 800-labeled antibody (KPL, USA) was carried out. Reactivity was detected and recorded on a Li-Cor Odyssey imaging system (Li-Cor Biosciences, USA).

### Transmission Electron Microscope (TEM)

The left lungs of the mice were fixed in 2.5% glutaraldehyde (Sigma, St. Louis, MO, USA) in PBS for 2 h. Then, the lungs were rinsed three times with PBS and exposed to 1% osmic acid (Sigma) for 2 h, followed by an additional three washes with PBS. Then, the lungs were dehydrated using an ascending alcohol series and infiltrated with Epon 812 overnight. The Epon 812-infiltrated tissues were placed in molds and polymerized at 80°C for 48 h. Ultrathin sections of the resulting blocks were cut to 60 nm using a Leica microtome and settled onto 400-mesh copper grids. The samples were stained for 15 min with 3% uranyl acetate and for 10 min in 1% lead citrate, followed by three washes with double-distilled water to take excess stain away. The samples were observed on a TEM (Hitachi H-7650, Japan).

### Pathologic and Histopathologic Analyses

The left lungs were immediately removed following euthanasia, and macroscopic lesions were photographed, after which the tissue was fixed overnight in 10% neutral buffered formalin. The lungs were dehydrated, embedded in paraffin, sectioned at 4 µm, stained with hematoxylin and eosin, and microscopically examined for histopathologic alterations and histopathologic scoring. The percentage of lung hemorrhage was scored as the following: 0 = no area; 1 <10%; 2 = 10–30%; and 3 >30% of lung surface. The severity of lung hemorrhage was scored from 0 to 3, where: 0 = absent; 1 = mild; 2 = moderate; and 3 = severe. The final injury score on the pathology scoring system was added according to the formula: severity of the lung hemorrhage + percentage of lung involvement. The percentage of lung hemorrhage was scored as the following: 0 = no area; 1 <10%; 2 = 10–30%; 3 = 30–50%; 4 = 50–70%; and 5 >70% of lung sections. The percentage of infiltration with inflammatory cells was scored from 0 to 4, where: 0 = no area; 1 <10%; 2 = 10–30%; 3 = 30–50%; and 4 >50% of the section area. The final histopathology score was determined according to the formula: percentage of lung hemorrhage + percentage of infiltration with inflammatory cells. The percentage of the lung surface was calculated by drawing square (specific calculation methods refer to Supplementary Material).

### Immunohistochemistry Assay

The paraffin-embedded lung tissues were deparaffinized and rehydrated. Sections were incubated in hydrogen peroxide to suppress endogenous activity and then were boiled in sodium citrate for antigen retrieval. Next, slides were incubated in normal goat serum to block the nonspecific binding of antibodies. Rabbit monoclonal anti-cleaved caspase-3 (Asp175) (D3E9) antibody (1:400 dilution, CST, Shanghai, China) was used as the primary antibody. Horseradish peroxidase-conjugated goat anti-rabbit IgG H&L (1:100 dilution, Abcam) was used as the secondary antibody. Immunoreactions were detected by DAB (Sigma-Aldrich), and the slides were counterstained with hematoxylin.

### Separation of Spleen Lymphocytes and Quantification of Inflammatory Indicators

The spleen was removed from the infected mice, and the spleen lymphocytes were separated using the Mouse Spleen Lymphocyte Separation Medium Kit (TBD Science, Tianjin, China) according to the manufacturer’s instructions. Separated lymphocytes and recombinant pMV262/MS, Rv0888NS/MS, D438A/MS, and H481N/MS were co-incubated overnight. IL-6, TNF-α, IL-1β, and IFN-γ levels in the supernatant were detected with a commercially available mouse IL-6, TNF-α, IL-1β, and IFN-γ ELISA Kit (eBioscience, Waltham, MA, USA) according to the manufacturer’s instructions.

### Statistical Analysis

All of the data are presented as mean ± SEM. One-way and two-way ANOVA were used to analyze statistical significance followed by Bonferroni’s multiple comparison test, and Prism software (version 5.0; GraphPad, San Diego, CA, USA) was used to carry out the analyses. A *P* value < 0.05 was considered to be statistically significant (*P < 0.05; **P < 0.01; ***P < 0.001).

## Results

### Analysis of DNase and Sphingomyelinase Activities of the Rv0888NS, D438A, and H481N Proteins

**Figure 1:**
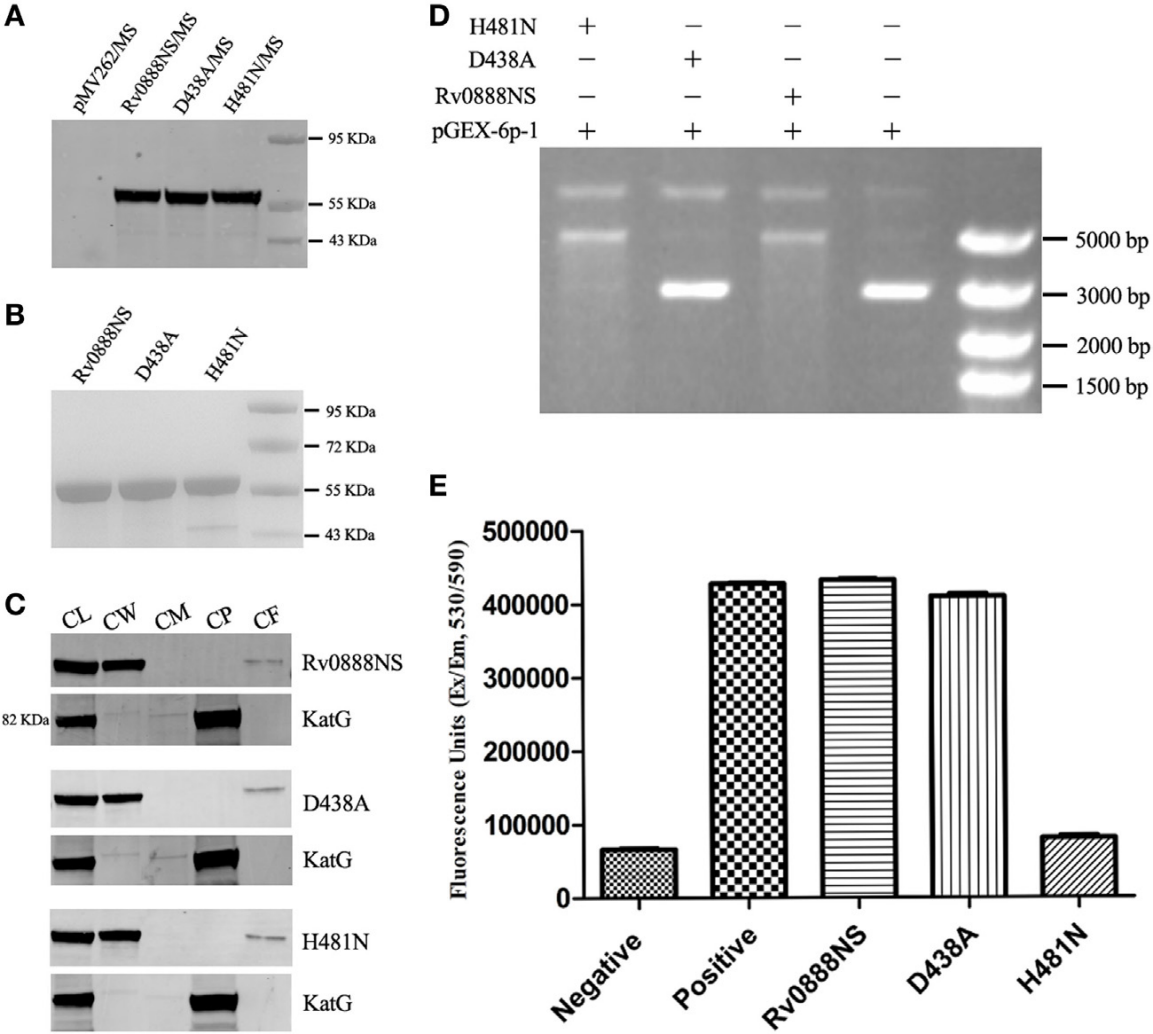
Activity analysis of the recombinant Rv0888NS, D438A, and H481N proteins. **(A)** Western blotting analysis of the expression of the Rv0888NS, D438A, and H481N proteins. **(B)** SDS-PAGE analysis of affinity-purified recombinant Rv0888NS, D438A, and H481N proteins. **(C)** Cell fractionation experiments were performed to determine the subcellular localization of Rv0888NS, D438A, and H481N; KatG protein served as a cytoplasmic marker for *Mycobacterium smegmatis*. CL represents whole-cell lysate proteins; CW represents cell wall; CM represents cell membrane; CP represents cytoplasm; and CF represents culture filtrate. **(D)** Assessment of DNase activity of the recombinant Rv0888NS, D438A, and H481N proteins. **(E)** Enzymatic sphingomyelinase assay using purified proteins; 20 µg of purified Rv0888NS, D438A, and H481N were analyzed using the Amplex Red Sphingomyelinase Kit. Sphingomyelin hydrolysis was determined using an excitation wavelength of 530 nm, and emission was detected at 590 nm. The error bars show the SEM of three independent experiments.

To investigate the DNase and sphingomyelinase activities of the Rv0888NS, D438A, and H481N proteins, we generated recombinant *M. smegmatis* strains. Rv0888NS, D438A, and H481N were successfully expressed in *M. smegmatis* (Figure 1A), obtained by Ni^2+^ affinity chromatography (Figure 1B), and localized to cell wall and culture filtrate components of *M. smegmatis* (Figure 1C). Purified Rv0888NS, D438A, and H481N were individually incubated with circular plasmid DNA. The results showed that circular plasmid DNA was degraded by the Rv0888NS and H481N proteins (Figure 1D). The Amplex Red Sphingomyelinase Assay Kit was used for determining the sphingomyelinase activity of the Rv0888NS, D438A, and H481N proteins. Rv0888NS and D438A showed clear sphingomyelinase activity, but H481N did not differ from the negative control (Figure 1E). These results indicate that the nuclease mutant (D438A) did not affect sphingomyelinase activity, and the sphingomyelinase mutant (H481N) also did not affect DNase activity. In other words, Rv0888NS has DNase and sphingomyelinase activity, D438A has sphingomyelinase activity, and H481N has DNase activity.

### Roles of Rv0888NS, D438A, and H481N in *M. smegmatis* Persistence in Lung Tissue

**Figure 2:**
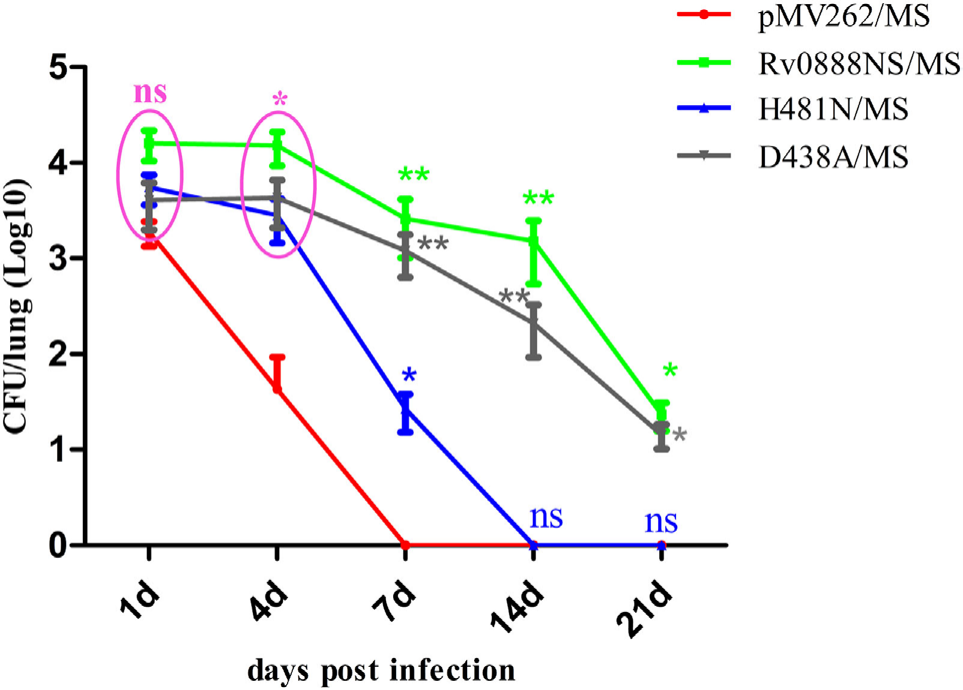
Presence of persistent recombinant *Mycobacterium smegmatis* in mouse lung. Bacterial loads in infected lung tissue from C57BL/6 mice, as transmitted by intraperitoneal infection with recombinant pMV262/MS, Rv0888NS/MS, D438A/MS, and H481N/MS were determined at 1, 4, 7, 14, and 21 days after infection (3 mice/group/time point). The error bars show the SEM of three independent experiments with three mice per group. ns, non-significant, *P < 0.05, **P < 0.01, two-way ANOVA followed by Bonferroni’s multiple comparison *post hoc* test.

To determine the roles of Rv0888NS, D438A, and H481N in *M. smegmatis* persistence in lung tissue, we constructed empty vector recombinant *M. smegmatis* strains (pMV262/MS), Rv0888NS recombinant *M. smegmatis* (Rv0888NS/MS), nuclease mutant recombinant *M. smegmatis* (D438A/MS), and sphingomyelinase mutant recombinant *M. smegmatis* (H481N/MS). Four groups of three mice were infected intraperitoneally with recombinant pMV262/MS, Rv0888NS/MS, D438A/MS, and H481N/MS, respectively. Bacterial loads in lung tissue were assessed at 1, 4, 7, 14, and 21 days after infection (Figure 2). There was no significant difference in the bacterial loads between the Rv0888NS/MS, D438A/MS, H481N/MS, and pMV262/MS groups at 1 day. The bacterial loads of Rv0888NS/MS and D438A/MS groups were dramatically higher compared with those of the pMV262/MS group at 4, 7, 14, and 21 days after infection, while the bacterial loads of H481N/MS group were not significant difference except 4 and 7 days after infection. As opposed to the almost entire clearance of bacteria in the lungs of the mice infected by pMV262/MS and H481N/MS groups at 7 and 14 days, respectively, bacterial loads sustained in the lungs of mice that were administrated with Rv0888NS/MS and D438A/MS at 21 days.

### Recombinant Rv0888NS/MS Sphingomyelinase Activity Induces NETs’ Formation *In Vivo*

**Figure 3:**
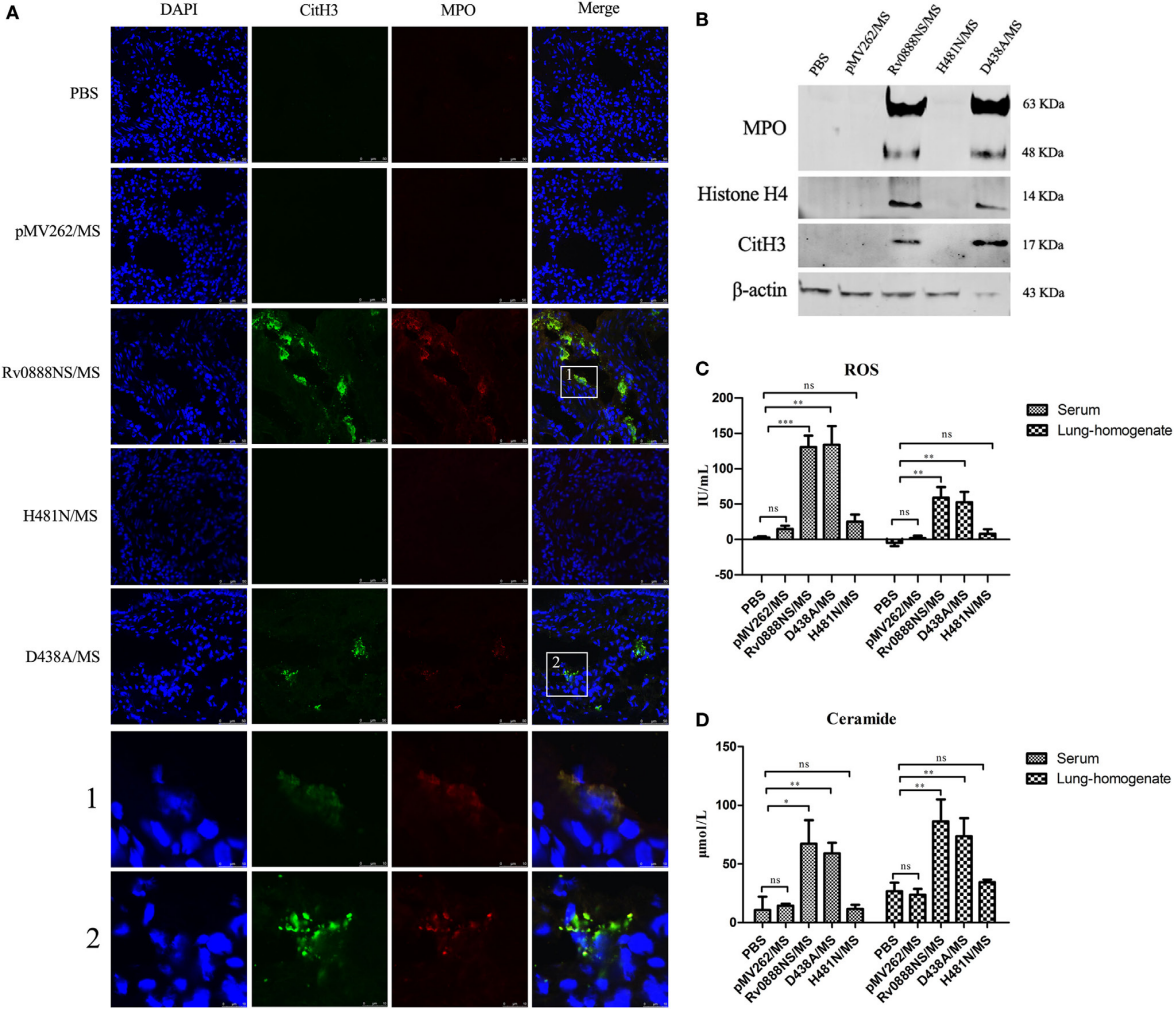
Recombinant Rv0888NS/MS sphingomyelinase induces neutrophil extracellular traps (NETs) formation in lung. **(A)** Immunofluorescence staining of lung sections from mice 48 h after intraperitoneal injection of recombinant Rv0888NS/MS, D438A/MS, and H481N/MS (*n* = 3 mice per group) administration was performed and compared with the control section PBS and recombinant pMV262/MS (*n* = 3 mice per group) for DNA (blue), CitH3 (green), and myeloperoxidase (MPO) (red). Images are representative of one of three independent experiments with three mice per group. The recombinant Rv0888NS/MS and D438A/MS groups’ merge showed co-localization of MPO with CitH3, indicating NETs’ formation. Scale bars = 50 µm. The higher magnification views of the insets (1 and 2), which were randomly chosen, show co-localization of DNA (blue), CitH3 (green), and MPO (red) in NETs structures. Scale bars = 10 µm. **(B)** Western blotting analysis of MPO, histone H4, and CitH3 protein levels in the bronchoalveolar lavage fluid. Images are representative of one of three independent experiments with three mice per group. **(C)** Reactive oxygen species (ROS) were measured in the serum and lung homogenates of infected mice using a Mouse ROS enzyme-linked immunosorbent assay (ELISA) Kit. **(D)** Ceramide in the serum and lung homogenates of infected mice was determined by the Mouse Ceramide ELISA Kit. The error bars show the SEM of three independent experiments with three mice per group. **(C,D)** One-way ANOVA followed by Bonferroni’s multiple comparison *post hoc* test, *P < 0.05, **P < 0.01, ***P < 0.001, ns, non-significant.

Various factors of pathogenic microorganisms, including α-enolase of *S. pneumoniae* (20), mycoplasma lipoproteins (21), and ESAT-6 of *M. tuberculosis* (3), can induce the formation of NETs. We investigated whether Rv0888-induced neutrophils to form NETs and determined if NETs’ formation was triggered by the nuclease or sphingomyelinase activity of Rv0888. We constructed a nuclease mutant recombinant D438A/MS and a sphingomyelinase mutant recombinant H481N/MS. Mice were injected intraperitoneally with recombinant Rv0888NS/MS, D438A/MS, and H481N/MS, followed by immunofluorescence staining of frozen acetone-fixed lung sections. NETs’ formation was detected in the lung tissues of mice administered recombinant Rv0888NS/MS and D438A/MS but not in the lung tissues of mice administered the recombinant H481N/MS. Control mice treated with PBS or recombinant pMV262/MS did not exhibit NETs’ formation. The formation of NETs was confirmed by staining for citrulline histone H3 (CitH3, green) and MPO (red) (Figure 3A), which are known NETs-associated proteins (34–36). In the following experiments, the levels of CitH3, MPO, and histone H4 were analyzed by Western blotting performed on the BALF of mice infected by recombinant pMV262/MS, Rv0888NS/MS, D438A/MS, and H481N/MS. In the Rv0888NS/MS and D438A/MS groups, CitH3, MPO, and histone H4 were clearly observed (Figure 3B). In the control groups (PBS and pMV262/MS) and the H481N/MS group, no corresponding proteins were detected (Figure 3B). The levels of ROS and ceramide were determined in serum and lung homogenates. In the Rv0888NS/MS and D438A/MS groups, the levels of ROS [the rise of ROS level promotes the formation of NETs (37)] and ceramide (the product of sphingomyelin degradation by sphingomyelinase) in the serum and lung homogenate were significantly greater than those in the control (PBS and recombinant pMV262/MS) and H481N/MS groups (Figures 3C,D). These data indicated that recombinant Rv0888NS/MS and D438A/MS can simulate the activity of sphingomyelinase in the mice, which induced NETs’ formation in the lung. The formation of NETs is likely to be dependent on ROS.

### Rv0888NS Sphingomyelinase Activity Induces NETs’ Formation *In Vitro*

**Figure 4:**
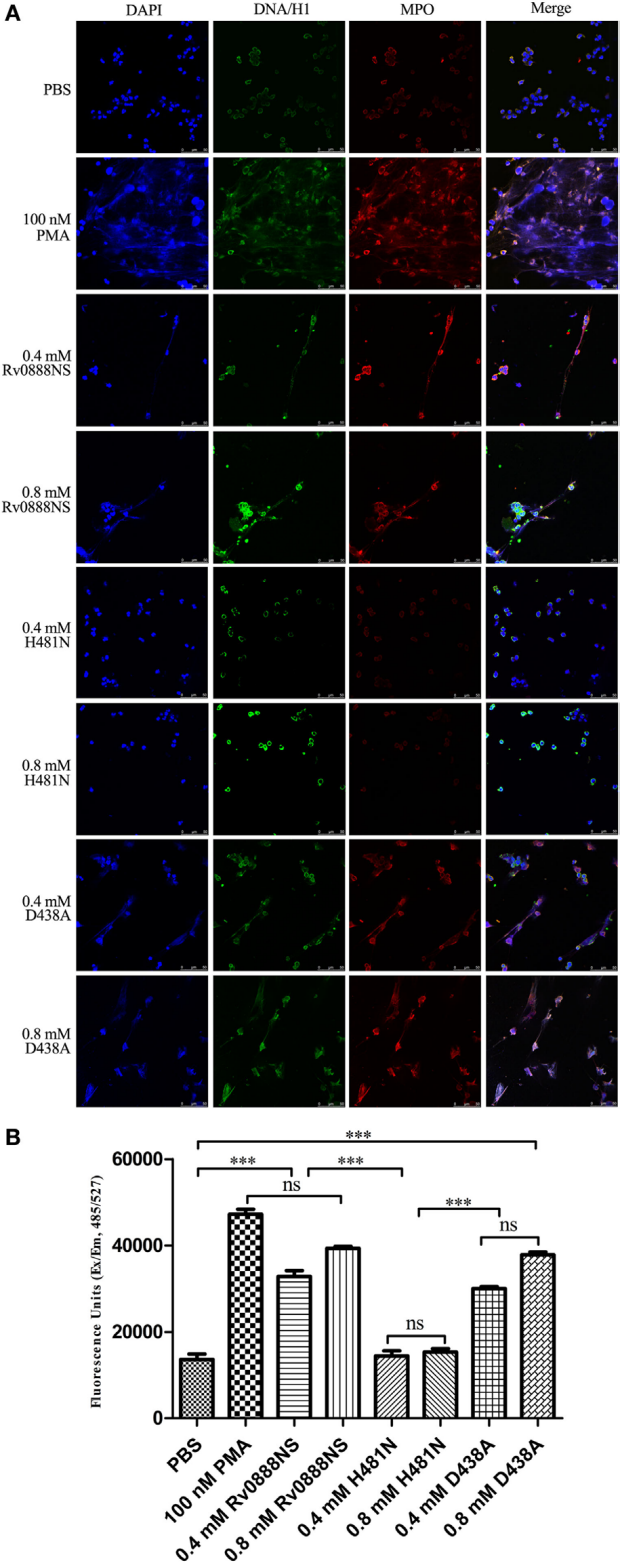
Rv0888NS sphingomyelinase induced formation of neutrophil extracellular traps (NETs) *in vitro*. **(A)** Human neutrophils were treated with 0.4 mM Rv0888NS or 0.8 mM Rv0888NS, 0.4 mM H481N or 0.8 mM H481N, and 0.4 mM D438A or 0.8 mM D438A. Alternatively, neutrophils were treated with 100 nM PMA (positive control) to induce the formation of NETs. Extracellular DNA was confirmed to be NETs by immunofluorescence microscopy by staining for DNA/H1 (green), myeloperoxidase (MPO; red), and DNA (DAPI; blue). Images are representative of one of three independent experiments with three wells per group. Scale bars = 50 µm. **(B)** Quantification of NETs release with the extracellular DNA stain Sytox green. The error bars show the SEM of three independent experiments with three wells per group. **(B)** One-way ANOVA followed by Bonferroni’s multiple comparison *post hoc* test, ***P < 0.001.

To investigate the ability of purified Rv0888NS, D438A, and H481N proteins to induce the release of NETs, human neutrophil cultures were established and administrated with PMA (positive control), PBS (negative control), 0.4 or 0.8 mM Rv0888NS, D438A, and H481N. As shown in Figure 4A, net-like structures were visible when neutrophils are incubated for 4 h with 0.4 mM Rv0888NS (third panel), 0.8 mM Rv0888NS (fourth panel), 0.4 mM D438A (seventh panel), and 0.8 mM D438A (eighth panel). The extracellular filamentous structures were stained for DNA (blue), DNA/histone H1 (green), and for MPO (red) to confirm that they were NETs. By contrast, NETs were never observed in 0.4 mM H481N (fifth panel), 0.8 mM H481N (sixth panel), and PBS-treated neutrophils (first panel). These results indicated that Rv0888NS sphingomyelinase works on neutrophils to induce NET formation.

We quantified the release of DNA from neutrophils with Sytox Green (Figure 4B). Activation of neutrophils with PMA (positive control), 0.4 mM Rv0888NS, 0.8 mM Rv0888NS, 0.4 mM D438A, and 0.8 mM D438A led to strong increases in extracellular DNA from 13,636 ± 2,168 to 32,841 ± 2,313, 39,323 ± 748, 30,033 ± 733, and 37,814 ± 1,141 FU, respectively. No significant change was observed between 0.4 mM H481N or 0.8 mM H481N-activated neutrophils and PBS-treated neutrophils.

### Recombinant Rv0888NS/MS Induces Lung Injury *via* Its Sphingomyelinase Activity

**Figure 5:**
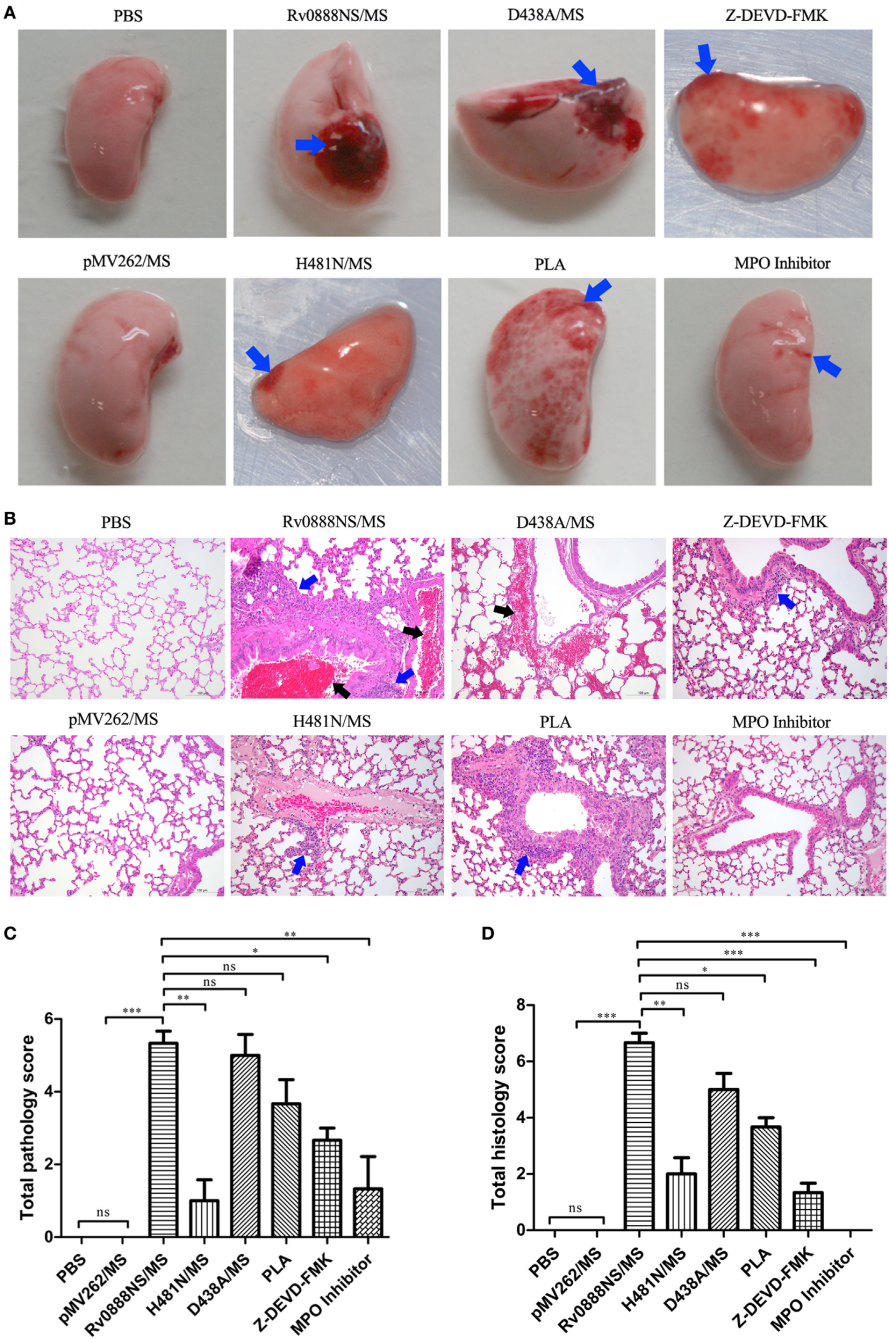
Macroscopic **(A)**, microscopic **(B)** lesion of lung and macroscopic lung lesion scores **(C)**, microscopic lung lesion scores **(D)** in infected mice by recombinant *Mycobacterium smegmatis*. C57BL/6 mice (*n* = 3 per group) were treated intraperitoneally with 2 × 10^6^ rMS (Rv0888NS/MS, D438A/MS, and H481N/MS). Control mice were treated with PBS and pMV262/MS. Co-injection of histone inhibitor [polysialic acid (PLA)], caspase-3 inhibitor (Z-DEVD-FMK), and myeloperoxidase (MPO) inhibitor with Rv0888NS/MS was carried out in the mice. After 48 h, the left lung was removed, the macroscopic lesions were photographically recorded and analyzed with HE staining. Images are representative of one of three independent experiments with three mice per group. **(A)** PBS and pMV262/MS: no pathological changes; Rv0888NS/MS: severe hemorrhage; H481N/MS: mild hemorrhage; D438A: severe hemorrhage; PLA: widespread petechial hemorrhage; Z-DEVD-FMK: small range of petechial hemorrhage; and MPO inhibitor: slight hemorrhage (blue arrow). **(B)** PBS and pMV262/MS: no pathological changes; Rv0888NS/MS: multiple inflammatory cells infiltration (blue arrow) and bronchiole cavity hemorrhage (black arrow); H481N/MS: small amount of inflammatory cell infiltration of around the local small vessel (blue arrow); D438A: hemorrhage of the partial alveolar wall and around the small vein (black arrow); PLA: widespread inflammatory cells infiltration around the bronchiole and small vessel (blue arrow); Z-DEVD-FMK: small amount of inflammatory cells infiltration around the bronchiole (blue arrow); and MPO inhibitor: no pathological changes. Scale bars = 100 µm. **(C,D)** One-way ANOVA followed by Bonferroni’s multiple comparison *post hoc* test, *P < 0.05, **P < 0.01, ***P < 0.001, ns, non-significant.

Previous research demonstrated that recombinant Rv0888NS/MS infection caused mild hyperplasia of alveolar epithelial cells in mice (31). To determine if these pathological injuries were caused by the nuclease or sphingomyelinase activity of Rv0888, the lungs were removed from infected mice for pathological investigations. The pathology results showed that lung hemorrhage in mice with recombinant H481N/MS was less than in mice infected with recombinant Rv0888NS/MS. However, hemorrhage in mice treated with recombinant D438A/MS was comparable with those treated with recombinant Rv0888NS/MS (Figure 5A). Histopathology results showed that infiltration of inflammatory cells into the lungs of mice infected by recombinant H481N/MS was reduced, and hemorrhage of the bronchiole cavity almost disappeared compared with mice administered recombinant Rv0888NS/MS (Figure 5B). There was no obvious difference in lung injury between mice administered D438A/MS and Rv0888NS/MS (Figure 5A). In addition, pathological and histopathological scores showed that there was a significant difference in injury-causing abilities between recombinant Rv0888NS/MS and H481N/MS but not between recombinant Rv0888NS/MS and D438A/MS (Figures 5C,D). These data demonstrate that recombinant Rv0888NS/MS induced lung injury in mice through its sphingomyelinase activity but not its nuclease activity.

### Ultrastructural Histopathology Studies of the Lung

**Figure 6:**
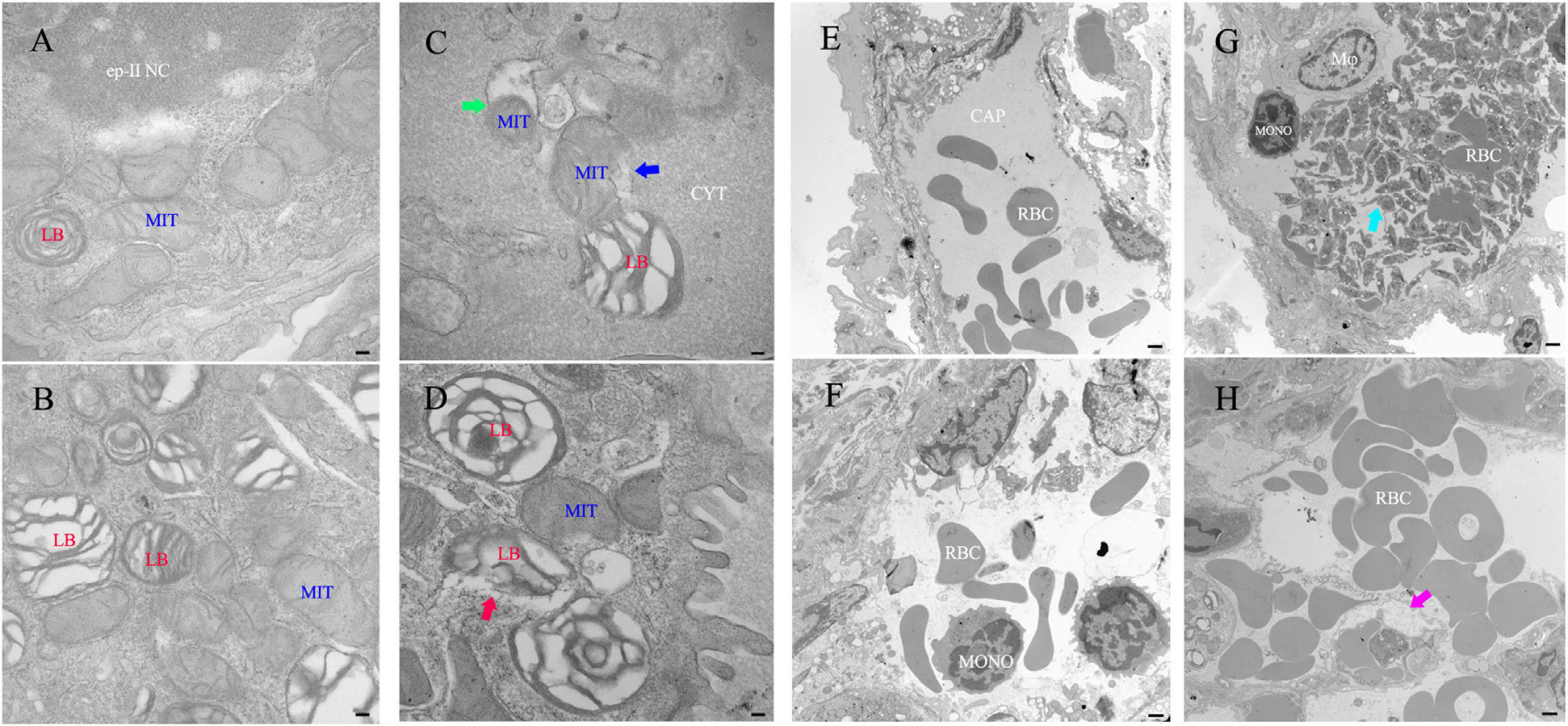
Transmission electron microscope histology of the lung in mice infected with recombinant *Mycobacterium smegmatis*. **(A–D)** Type II pneumocyte. **(E,F)** Small vein. **(A,E)** PBS. **(B,F)** pMVp262/MS. Rv0888NS/MS-treated mice (*n* = 3 per group) show that the number of mitochondrial cristae decreased, ruptured, and degenerated **(C)**, blue arrow and mitochondrial autophagy **(C)**, green arrow, ruptured membrane of the lamellar body **(D)**, red arrow; platelet-rich microthrombi **(G)**, cyan arrow; small blood vessels edema **(H)**, purplish-red arrow. Images are representative of one of three independent experiments with three lung sections per group. Abbreviations: RBC: red blood cells; LB: lamellar body; MIT: mitochondrial; Mφ: macrophage; ep-II NC: type II pneumocyte nuclear chromatin; CYT: cytoplasm; CAP: capillary; MONO: monocytes. Scale bars: **(A–D)** 0.5 µm; **(E,F)**, 5 µm.

For ultrastructural histopathology studies of the lung, the left lung was removed from infected mice (administered recombinant Rv0888NS/MS) and control mice (administered PBS or recombinant pMV262/MS), and ultrathin sections were observed using a TEM. Recombinant Rv0888NS/MS-treated mice showed impairment of organelles in type II pneumocytes, including a decreased number, rupture, and degeneration of mitochondrial cristae (Figure 6C, blue arrow); mitochondrial autophagy (Figure 6C, green arrow); and membrane rupture of the lamellar body (Figure 6D, red arrow). Platelet-rich microthrombi in the small vein (Figure 6G, cyan arrow) and small blood vessel edema (Figure 6H, purplish-red arrow) were observed. By contrast, impairment of organelles of type II pneumocytes, platelet-rich microthrombi in the small veins, and small blood vessel edema were never observed in the PBS-treated (Figures 6A,E) and recombinant pMV262/MS-administrated mice (Figures 6B,F).

### Recombinant Rv0888NS/MS Sphingomyelinase Regulates the Inflammatory Response

**Figure 7:**
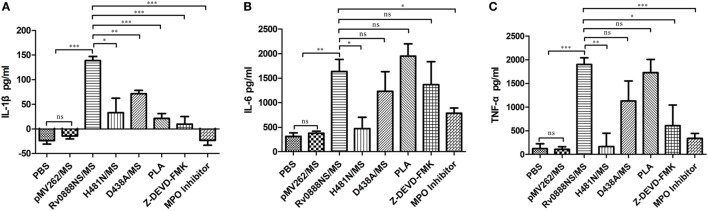
Quantification of inflammatory indicators in mice infected with recombinant *Mycobacterium smegmatis*. The spleen was excised from the infected mice (*n* = 3 per group), and the spleen lymphocytes were separated and incubated overnight with PBS, pMVp262/MS and Rv0888NS/MS, D438A/MS, and H481N/MS. The cytokines were determined using the cultured supernatant. **(A)** Analysis of the IL-1β. **(B)** Analysis of IL-6. **(C)** Analysis of TNF-α. The error bars show the SEM of three independent experiments with three wells per group. **(A–C)** One-way ANOVA followed by Bonferroni’s multiple comparison *post hoc* test, *P < 0.05, **P < 0.01, ***P < 0.001, ns, non-significant.

To quantify the degree of inflammation, levels of the cytokines IL-6, TNF-α, and IL-1β were measured in the supernatant of stimulated spleen lymphocytes (Figure 7). IL-6, TNF-α, and IL-1β levels in mice treated with recombinant Rv0888NS/MS were significantly higher than those in control mice (PBS and recombinant pMV262/MS). IL-1β levels in mice with recombinant D438A/MS and IL-6, IL-1β and TNF-α levels in mice treated with recombinant H481N/MS were markedly reduced compared with mice administered recombinant Rv0888NS/MS. These data showed that the inflammatory reaction was mainly regulated by sphingomyelinase and partly by nuclease.

### Recombinant Rv0888NS/MS Induces Lung Injury and Inflammation *via* MPO and Histones in NETs

Based on the previous results, we speculated that NETs’ formation may be correlated with the inflammatory response and lung injury. MPO activity can induce injuries in adjacent tissue and favoring numbers of inflammatory diseases, including lung lesion (26, 27). Pre-incubation of NETs with PLA considerably reduced NET-mediated cytotoxicity of A549 cells (25). Based on this assumption and our previous study, the mice were treated with an MPO inhibitor and PLA. Injection of the MPO inhibitor in mice administered Rv0888NS/MS led to a reduction in Rv0888-induced pathological and histopathological lesions (Figures 5A,B). Pathologic and histopathologic scores showed very significant differences between Rv0888NS/MS and the MPO inhibitor (Figures 5C,D). IL-6, TNF-α, and IL-1β levels after treatment with the MPO inhibitor were significantly lower than in mice treated with Rv0888NS/MS alone (Figures 7A–C). Injection of PLA in mice challenged with Rv0888NS/MS led to a slight decrease in Rv0888-induced histopathologic changes but no change in pathology (Figures 5A,B). IL-1β levels after PLA treatment were markedly reduced compared with those in mice administered Rv0888NS/MS alone (Figure 7A). However, there were no significant differences in the levels of IL-6 and TNF-α in PLA-treated mice (Figures 7B,C). These data demonstrate that Rv0888NS/MS-induced inflammation and injury of the lung is partly mediated by histone, in addition to MPO.

### MPO-Mediated Lung Injury and Inflammation *via* Activation of Caspase-3

**Figure 8:**
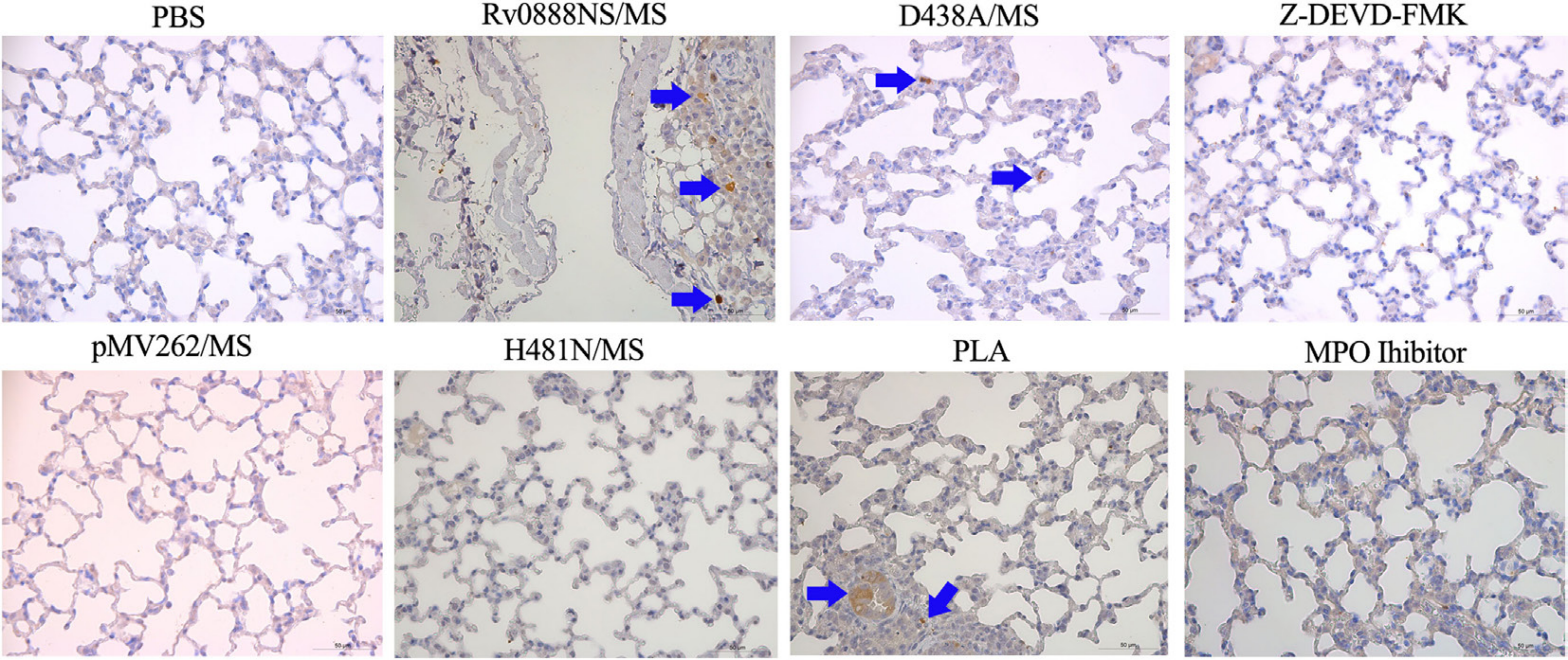
Immunohistochemical analysis of the lung sections in mice infected with recombinant *Mycobacterium smegmatis*. Paraffin-embedded sections of lung tissues were examined by immunohistochemistry with cleaved caspase-3 (ASP175) antibody {cleaved caspase-3 stained as brown pigment (blue arrows) [Rv0888NS/MS, D438A/MS, and polysialic acid (PLA)]} counterstained with hematoxylin. Images are representative of one of three independent experiments with three lung sections per group. Scale bars = 50 µm.

The previous results indicated that recombinant Rv0888NS/MS-induced lung injury is largely mediated by MPO. MPO can stimulate caspase-3 activation-mediated apoptosis in HL-60 human leukemia cells (38). Immunohistochemical expression of cleaved caspase-3 was used as a marker of activation in the lung tissues isolated after intraperitoneal injection of recombinant *M. smegmatis* into C57BL/6 mice. Caspase-3 activation was observed by immunohistochemical staining in the lungs of mice administered recombinant Rv0888NS/MS, D438A, and PLA (Figure 8). Caspase-3 staining was not observed in lung tissue from MPO inhibitor groups (Figure 8). These results indicate that MPO-mediated lung injury and inflammation occurs *via* activated caspase-3.

### Inhibition of Caspase-3 Reduces Recombinant Rv0888NS/MS-Induced Lung Injury and Inflammation

To confirm the importance of caspase-3 in lung injury and inflammation, the caspase-3 inhibitor, Z-DEVD-FMK, was co-injected with recombinant Rv0888NS/MS. The introduction of Z-DEVD-FMK reduced recombinant Rv0888NS/MS-induced pathologic and histopathologic changes (Figures 5A,B), and pathologic and histopathologic scores showed that the difference between Rv0888NS/MS and Z-DEVD-FMK was significant (Figures 5C,D). Caspase-3 was not detected in the lungs of mice treated with Z-DEVD-FMK (Figure 8), and IL-1β and TNF-α levels were markedly reduced compared with mice administered recombinant Rv0888NS/MS (Figures 7B,C). These data again confirm that MPO-mediated lung injury occurred *via* activated caspase-3.

## Discussion

Various bacterial sphingomyelinases are critical for the virulence of extracellular, facultative, or intracellular pathogenic microorganisms. *B. cereus* Sphingomyelinase C causes colon epithelial cell death and serves to the virulence of *B. cereus* in a *Galleria mellonella* larval infection model (39–41). *Listeria ivanovii*, a Gram-positive facultative intracellular pathogen of ruminants, produces a sphingomyelinase that is involved in the damage of the subsequent intracellular proliferation and phagocytic vacuole (42). Comparing with the wild-type strain, the intracellular proliferation capacity of a sphingomyelinase knockout strain of *L. ivanovii* possessed a reduced ability to propagate intracellularly and was less virulent in mice (42). But there are no data on sphingomyelinase as a virulence factor in the *M. tuberculosis*. In our study, we successfully constructed the Rv0888NS/MS recombinant *M. smegmatis* and identified Rv0888 sphingomyelinase not only promotes colonization of the *M. smegmatis* in the lung but also induces lung lesion in the mice. These results implicate that Rv0888 sphingomyelinase is adequate to confer pathogenic characters to *M. smegmatis* and may be a virulence factor of *M. tuberculosis*.

Owning to the intracellular infections of mycobacteria, little attention has been paid to the potential extracellular function, which neutrophils might play a role in TB. Neutrophils were infiltrated in the intra-alveolar of the mice infected by *M. tuberculosis* and can aggravate the lung lesions induced by *M. tuberculosis* (43, 44). Dallenga et al. find that neutrophils infected by *M. tuberculosis* induce necrotic cell death, which contributes bacterial growth while engulfed by macrophages (45). And *M. tuberculosis* induced NETs’ formation *in vitro* and trapped mycobacteria, but the NETs were unable to kill the bacteria (29, 30). The inability of NETs to kill mycobacteria may favor lung destruction in active TB. Here, we describe the *in vivo* formation of NETs in lungs of mice infected by recombinant Rv0888NS/MS and D438A/MS. NETs that were observed in the lung tissue lacked mesh-like extracellular chromatin structures, possibly due to the space limitations of the lung parenchyma (46). These results identify previous discoveries in which NETs-related inflammatory protein was observed in the lung granulomas of patients with active TB (47). Therefore, NETs could be involved in the formation of tissue injury related to inflammatory disease that is caused by mycobacteria. Studies on the ability of Rv0888 to induce NETs were initially made *in vitro* by stimulating human blood neutrophils with either purified Rv0888NS or D438A and H481N proteins. The results indicated that only the sphingomyelinase activity of Rv0888NS efficiently induces NETs’ formation, whereas NETosis does not occur upon exposure of neutrophils to its nuclease activity.

Transmission electron microscope results showed that the membrane of lamellar bodies in type II pneumocytes was ruptured (Figure 6D). The lamellar body is unique to type II pneumocytes, which are lined with a pulmonary surfactant layer secreted by lamellar-containing surfactant proteins, A (SP-A) and D (SP-D), which help protect the lungs from inflammation and infection (48). The binding of these surfactant proteins to apoptotic cells and DNA improves their clearance through alveolar macrophages (49, 50). SP-D, in particular, has a function in decreasing the apoptosis of pro-inflammatory cytokines and alveolar macrophages (51). Thus, the loss of function of the lamellar body is suggested to affect the anti-infection and anti-inflammation properties of the lung. We observed that mitochondrial cristae decreased, ruptured, and degenerated (Figure 6C). Mitochondria are the main sites of biological oxidation and energy conversion of eukaryotic cells, and 80% of the energy required for cellular activities is provided by this organelle. Damage to mitochondria usually results in a decrease of ATP energy and an increase in ROS and lactic acidosis, the latter of which can cause cell damage or apoptosis (52).

We observed NETs’ formation in the lung (Figure 3A) and the aggregation of platelets (Figure 6G) in the lung capillaries. NETs promote platelet binding and aggregation with a scaffold and stimulus, and the administration of platelets with purified histones is adequate for aggregation (53). Extracellular histones promote platelet-dependent thrombin generation (54). CitH3 and histone H4 were detected in the BALF (Figure 3B). It is possible that the observed aggregation of platelets in the lung capillaries was caused by extracellular histone. Platelets are major contributors to damage and acute inflammation (55) in conditions like rheumatoid arthritis (56) and cerebral malaria (57) as well as acute lung lesion (58).

Neutrophils were confirmed as the primary immune cell type in BALF and sputum from active TB patients (59). *M. tuberculosis* can induce neutrophils to release NETs coated with neutrophil elastase and histones (29). In addition, sputum induced by *M. tuberculosis* collected from TB patients had a increased number of NETs compared with healthy individuals (60). ESAT-6 secreted by *M. tuberculosis* induces the production of NETs colocalized with MPO (3). In this study, MPO, CitH3 and histone H4 were detected in the lung BALF (Figure 3B). MPO, a granular protein of NETs, makes DNA strand disrupted in lung epithelial cells (61) and contributes to the NET-mediated cytotoxicity of epithelial cells (25). Also, interference with histones by antibodies, PLA, or activated protein C can decrease NET-mediated cytotoxicity (25, 28). Co-injection of recombinant Rv0888NS/MS with an MPO inhibitor remarkably decreased recombinant Rv0888NS/MS-induced pulmonary hemorrhage, infiltration of inflammatory cells, and release of inflammatory cytokines (IL-6, TNF-α and IL-1β). Co-injection of recombinant Rv0888NS/MS with PLA, which had been identified as being a binding partner for histone, slightly decreased the histopathologic changes. These data indicate that the inflammation and pathologic injury induced by recombinant Rv0888NS/MS was possibly mediated by NETs, which trapped mycobacteria but did not kill them as determined by a previous *in vitro* study (30). This inability of NETs to kill mycobacteria may contribute to the lung destruction that occurs during *M. tuberculosis* infection.

Neutrophil extracellular traps induced by *M. tuberculosis* stimulate the release of high levels of IL-6, TNF-α, and IL-1β from macrophages (30). We showed that NETs-mediated lung injury promoted the release of the inflammatory cytokines IL-6, TNF-α and IL-1β. In acute inflammatory responses, neutrophils and neutrophil-derived cytokines are participated in the pathogenesis of chronic inflammatory disorders, such as mycobacterial infections, inflammatory bowel diseases and rheumatoid arthritis (62). Some of these cytokines (e.g., TNF-α) take part in recruiting more neutrophils or other leukocytes to the site of sterile inflammation or infection (63). The recruitment of neutrophils to infected lung is negatively influenced by IFN-γ, which is pro-inflammatory and inhibits the accumulation of neutrophils (64). However, our data showed that IFN-γ levels were not increased in recombinant Rv0888NS/MS-infected mice compared with PBS- and recombinant pMV262/MS-treated mice (Figure S1 in Supplementary Material). This may lead to amounts of neutrophils being recruited to the site of infection, thereby aggravating the inflammatory response and lung injury. Myzak et al. (36) showed that H_2_O_2_-induced caspase-3 activation in HL-60 cells *in vitro* is MPO-dependent. This is the first study to show that Rv0888-induced caspase-3 activation in lung tissue is also MPO-dependent *in vivo*, and mitophagy in the type II pneumocytes of the lung was observed. It is possible that Rv0888-induced injury and inflammation of the lung were caused by the mitochondrial pathway and extrinsic signaling pathways that initiated apoptosis.

In summary, we demonstrated that sphingomyelinase activity of the recombinant Rv0888NS/MS enhances the colonization ability of *M. smegmatis* in the lungs of mice and induces NETs’ formation in the lungs of infected mice; causes hemorrhage and infiltration of inflammatory cytokines; and activates caspase-3. The protein components of the formed NETs were mainly MPO, histone H4 and CitH3. Inhibition of either MPO or caspase-3 significantly reduced lung injury and the release of inflammatory cytokines, and inhibition of histone-induced bronchial hemorrhage. These results show that NETs, induced by Rv0888 sphingomyelinase, play an important role in the development of tissue injury concerning with acute inflammatory disease caused by mycobacterium.

## Ethics Statement

All of the experimental work that involved animals was carried out following the instructions that were suggested by the Heilongjiang Animal Ethics Committee at the Heilongjiang science and technology government agency (Harbin, China) and was approved and supervised by the Commissioner for Animal Welfare at the Harbin Veterinary Research Institute, representing the Institutional Animal Care and Use Committee. All of the experimentations were conceived to minimize the numbers of animals that were used, and effort was made to minimize both distress and their pain.

## Author Contributions

GD, NS, LC, HP, and SL conceived and designed the study. GD and SL wrote the manuscript. GD and YC performed the study. LW, TL, and ZC provided research protocols and reagents. All of the authors have read and agreed with the data.

## Conflict of Interest Statement

The authors announce that the study was carried out in the absence of any commercial or financial relationships that could be construed as a potential conflict of interest. The reviewer TD and handling Editor declared their shared affiliation.
