# Supplementary material for: Extracellular Sphingomyelinase Rv0888 of Mycobacterium tuberculosis Contributes to Pathological Lung Injury of Mycobacterium smegmatis in Mice via Inducing Formation of Neutrophil Extracellular Traps
Source: Front Immunol. 2018 Apr 4;9:677. doi: 10.3389/fimmu.2018.00677 (PMC5893642; doi:10.3389/fimmu.2018.00677)
Supplement: Supplementary file 1 [file image_1.PDF]

Supplementary Figure

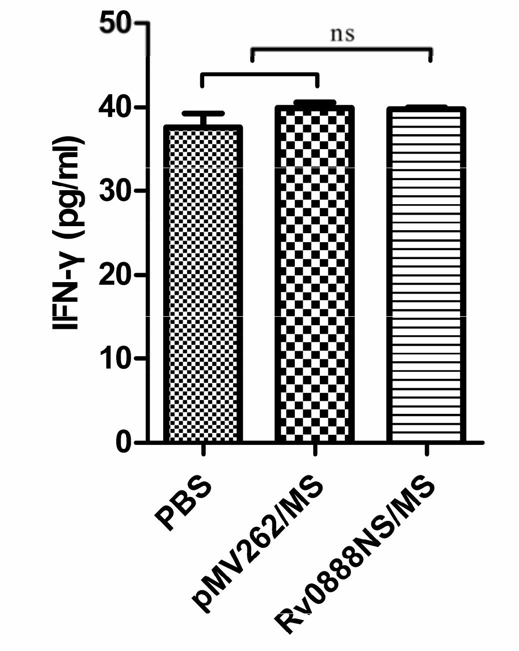

Supplementary Figure 1. Analysis of inflammatory cytokine IFN-γ

The spleen was excised from PBS-, recombinant pMVp262/MS-, and Rv0888NS/MS-administered mice, and the spleen lymphocytes were separated and incubated overnight with PBS, recombinant pMVp262/MS, and Rv0888NS/MS, respectively. There was no difference in IFN-γ levels between the recombinant Rv0888NS/MS and the controls (PBS and recombinant pMV262/MS).
