# Supplementary material for: Extracellular Sphingomyelinase Rv0888 of Mycobacterium tuberculosis Contributes to Pathological Lung Injury of Mycobacterium smegmatis in Mice via Inducing Formation of Neutrophil Extracellular Traps
Source: Front Immunol. 2018 Apr 4;9:677. doi: 10.3389/fimmu.2018.00677 (PMC5893642; doi:10.3389/fimmu.2018.00677)
Supplement: Supplementary file 2 [file table_1.doc]

**Supplementary Information**

**Supplementary Tables**

Table S1. The complementary mutagenic oligonucleotides used in this study.

| Primer | Sequence 5’ 3’ |
| --- | --- |
| D438A-F | CGGCAACGAGTGCGAGCTGCTCGCCAAGAT |
| D438A-R | CCGCTTCGATAGAAGATCTTGGCGAGCAGC |
| H481N-F | CAGCCCGGCGGTGGTCGGCTTCAACTACGT |
| H481N-R | GCCACGTTGTCCGCGACGTAGTTGAAGCCG |
